# Safety and Efficacy of a New Endocapsular Device Used in Age-Related Cataract Surgery: Twelve-Month Follow-Up

**DOI:** 10.1167/tvst.15.2.8

**Published:** 2026-02-06

**Authors:** Ioannis G. Pallikaris, Ahmed Elmassry, Harilaos S. Ginis, Loukia L Leonidou, Onurcan Sahin, Manolis Modatsos, Dimitris Liakopoulos, Shaimaa Elbassiouny, Aristofanis Pallikaris

**Affiliations:** 1 Laboratory of Optics and Vision, Medical School, University of Crete, Heraklion, Greece; 2 Ophthalmology Department, Faculty of Medicine, Alexandria University, Alexandria, Egypt; 3 El-Nour Eye Center, Alexandria, Egypt; 4 Department of Mechatronics, Yıldız Technical University, Istanbul, Turkey

**Keywords:** endocapsular device, cataract surgery, posterior capsular opacification, refractive stability, intraocular lens

## Abstract

**Purpose:**

To evaluate the safety and efficacy of a novel endocapsular device used during cataract surgery at the 12-month follow-up.

**Methods:**

A cohort study was conducted in a university-affiliated private practice. Adults with age-related cataracts, intraocular lens power between 14 and 26 diopters, and no other ocular pathology were included prospectively. Exclusion criteria included diabetes, previous ocular surgery, cardiac conditions, and autoimmune diseases. One eye per patient was randomly assigned to receive the device before intraocular lens implantation. Follow-ups were conducted preoperatively and at 1 day, 1 week, and 1, 3, 6, and 12 months postoperatively. The primary safety endpoint was the incidence of adverse events; the primary efficacy endpoint was the incidence of posterior capsule opacification (PCO) postoperatively at 12 months. A control group was formed retrospectively for comparison of PCO at 12 months. Spherical equivalent, corrected distance visual acuity, and intraocular pressure were also measured.

**Results:**

A total of 121 patients were enrolled. Sixteen adverse events occurred in 12 patients; all resolved and were deemed unrelated to the device. PCO incidence at 12 months was 0.83% in the experimental group vs. 13.0% in the control group. The spherical equivalent stabilized by 3 months. At 12 months, the mean corrected distance visual acuity and intraocular pressure were 0.03 ± 0.07 logarithm of the minimum angle of resolution and 11.23 ± 2.03 mm Hg, respectively.

**Conclusions:**

The new device seems to be safe and have a beneficial impact on PCO up to at least 12 months postoperatively.

**Translational Relevance:**

We report our experience with a novel, safe, endocapsular open capsule device that shows promise in preventing posterior capsule opacification in real-world clinical settings.

## Introduction

Replacement of the human natural crystalline lens with an intraocular lens (IOL) implant to treat cataract, presbyopia, or high refractive error is one of the most common and safest surgical procedures performed worldwide.^1^^–^^3^ Despite the overall success of these procedures and the advancements in surgical techniques and IOL designs, such as square-edge designs and improved materials,^4^^–^^6^ a number of patients fail to achieve optimal visual outcomes owing to various postoperative complications.

A key limitation of current lens replacement techniques is the failure to maintain the natural three-dimensional structure of the capsular bag and its intracapsular space. This results in capsular bag contraction/shrinkage and postoperative capsular fibrosis, which can cause complications such as posterior capsule opacification (PCO) or late IOL opacifications, capsular phimosis, some uncertainty in the actual effective lens position, and IOL dislocation or tilt. Together, these factors can significantly impair the achievement of optimal visual outcomes.^7^^–^^11^ PCO, in particular, remains a persistent issue. Even though PCO may be treated with neodymium-doped yttrium aluminum garnet (Nd:YAG) laser capsulotomy, this treatment carries additional risks, such as retinal detachment, increased intraocular pressure (IOP), and IOL damage.^12^

To counteract these structural changes and instability of the capsular bag, various endocapsular devices such as capsular tension rings (CTRs) have been developed, particularly for cases involving zonular weakness.^13^^–^^17^ CTRs in particular help to maintain the circular contour of the capsular bag and balance zonular tension, thereby decreasing the risks of bag shrinkage and IOL dislocation. However, CTRs do not completely restore the natural anatomy of the lens capsule, nor do they entirely and effectively prevent PCO. In addition, special types of IOLs and intracapsular devices that seem to maintain the capsular bag open have been developed. Experimental and clinical studies have reported that these devices are associated with lower rates of PCO.^18^^–^^24^ These findings have led to the hypothesis that preserving the openness of the capsular bag could help to prevent PCO. Despite the promising results, these special types of devices have not been evaluated extensively in large-scale clinical trials or integrated into routine clinical practice.

Recently, we reported a case using a new implantable endocapsular device developed to preserve the natural three-dimensional shape of the capsular bag and partially refill the intracapsular space and complement cataract and refractive lens exchange surgery.^25^ Given the growing evidence that preserving capsular bag shape may decrease PCO, this study aimed to primarily evaluate the safety—measured by the incidence of adverse events (AEs)—and the efficacy of this new device in preventing PCO over a 12-month period in a cohort of patients undergoing cataract surgery. As a secondary aim, the study evaluated the basic clinical evaluation metrics associated with the implantation of the device.

## Methods

### Design

Patients who presented for standard cataract surgery in a university-affiliated private clinical practice between December 2019 (first patient, first visit) and June 2022 (last patient, last visit) were enrolled prospectively in this cohort clinical investigation. From all eligible patients attending the clinic, every third patient who met the inclusion and exclusion criteria was prospectively enrolled in the experimental group. The study adhered to the tenets of the Declaration of Helsinki and was approved by the Institutional Ethics Committee of Alexandria University (Approval Number: 0304502). All participants signed a written informed consent before undergoing any intervention. The clinical trial was registered with the Pan African Clinical Trials Registry database (https://pactr.samrc.ac.za/) with identification number PACTR202106741373163. Clinical records of 100 patients who had undergone standard cataract surgery during the same time period, in the same private clinic, and by the same surgeon but who were not enrolled in the experimental group were used to form a control group retrospectively. Similar to the experimental group, every third eligible data file was selected and the patient was asked to sign a written informed consent for access to their medical relevant data. Unlike the experimental group, the control group was formed solely to assess the 1-year postoperative incidence of PCO.

### Patients

Adhering to the guidelines specified in ISO 11979-7, appendix D.8.2, the sample size was defined to be approximately 120 subjects. Patients were included in the study if they fulfilled the following criteria: (a) adult males and females with age related cataract, (b) suitable to receive a standard monofocal IOL with power ranging from +14 to +26 diopters, (c) clear intraocular media aside from cataract, (d) nuclear cataract grade between N1 and N3 according to the Lens Opacities Classification System III, (e) no history of intraocular or corneal surgery, (f) no traumatic cataract, (g) stable keratometry and biometry measurements, with no signs of corneal ectasia or corneal dystrophies, (h) no irregular astigmatism, (i) no pregnancy or lactating potential, and (j) no concurrent participation in another drug or device investigation. Exclusion criteria were (a) dense cataract, (b) presence of additional anterior segment pathology (e.g., glaucoma, pseudoexfoliation syndrome), (c) prior corneal surgeries (LASIK, photorefractive keratectomy, etc.), (d) monocular patient, and (e) inability to attend postoperative follow-up visits. Criteria and procedures for subject withdrawal or discontinuation were capsule rapture, zonular dehiscence, and retained lens matter. To form the control group, data were collected from patients that adhered to the same inclusion and exclusion criteria.

### Surgical Procedure

**Figure 1.**
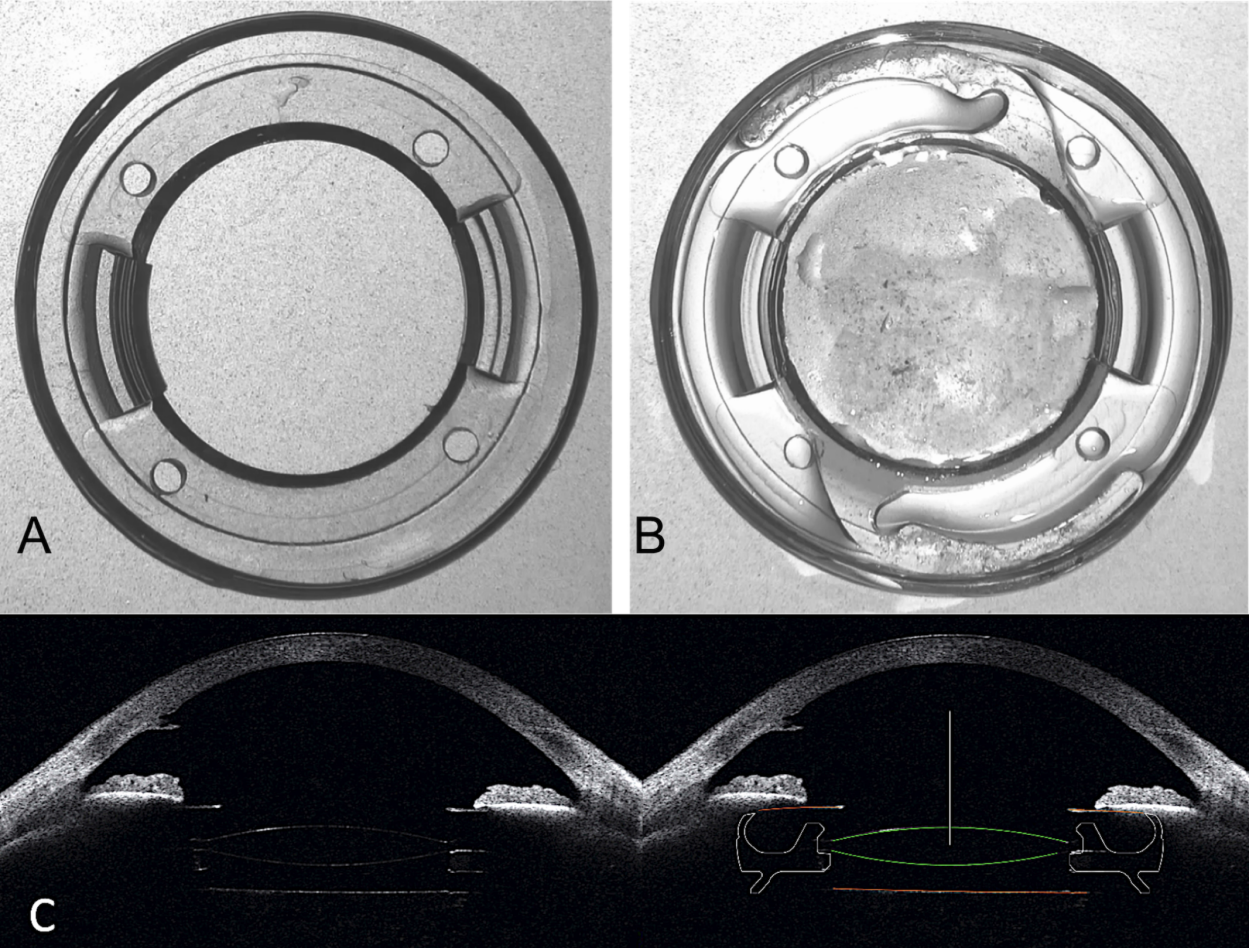
(**A**) New endocapsular device. (**B**) New endocapsular device with a single piece IOL. (**C**) Scheimpflug image of eye with new endocapsular device and IOL in place, and conceptual sketch of the location of the new endocapsular device and the IOL in the capsule after cataract surgery.

The patients were implanted with the new intraocular implant (fixOflex, EYE PCR BV) in one eye. Eye selection was randomized using a custom MATLAB algorithm (MATLAB R2017b, MathWorks, Natick, MA) using the function “randsample.” The medical device was specifically designed by taking into account the anatomical dimensions of the capsule. The intraocular implant is made of hydrophilic acrylic prepolymerized material with an overall diameter of 9.80 mm and a central opening sufficient to accommodate any IOL with a 6-mm optic. Its overall thickness is 1.84 mm, its weight is 32.5 mg, and it maintains circumferential contact with the capsular bag. In addition, it features structural elements to stabilize the IOL's optical part (Fig. 1).

**Figure 2.**
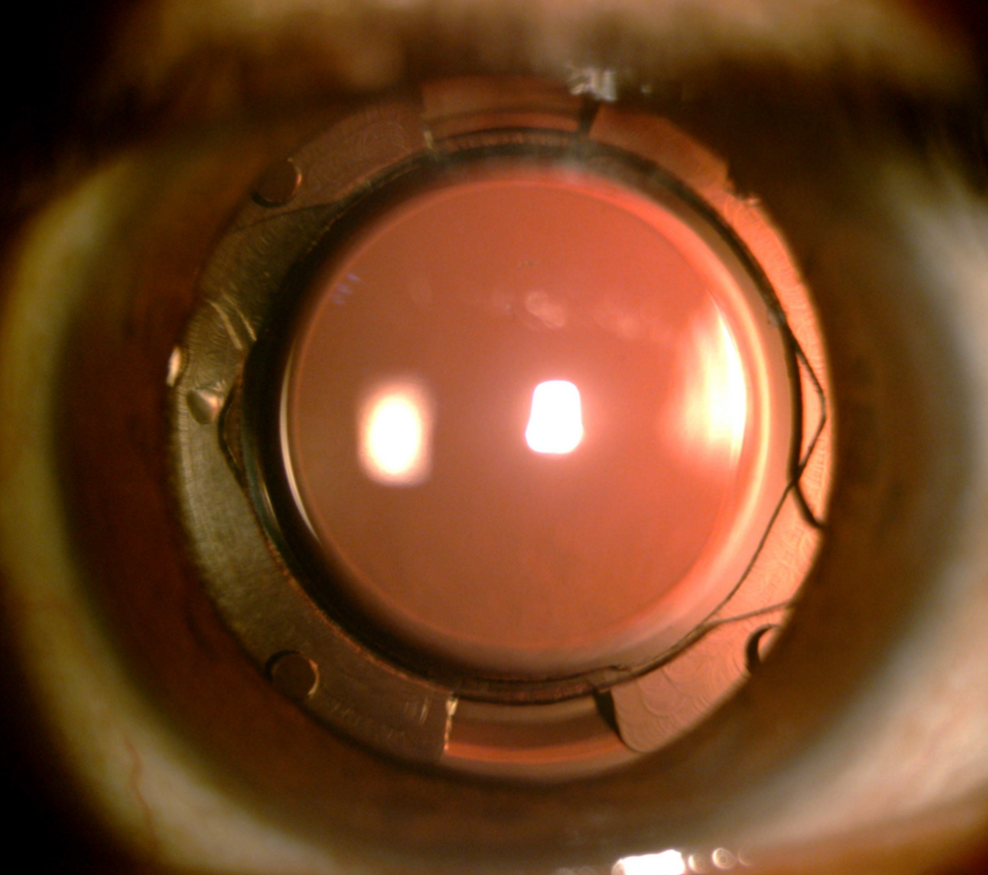
Slit lamp photograph, 12 months postoperatively, showing the IOL in the new endocapsular device.

The intraocular endocapsular device was implanted in the bag at the presence of viscoelastic using a 2.4-mm injector through a standard capsulorhexis (intended size of 6 mm), following standard phacoemulsification and before IOL implantation. After expansion of the intraocular implant in the capsule, all patients received the same type of IOL (Tecnis ZCB00, Johnson & Johnson Vision, Jacksonville, FL) inserted into the central aperture of the device (Fig. 2; Supplementary Video S1). The dioptric power of each IOL was calculated based on standard biometry (LS 900 Lenstar, Haag-Streit, Koniz, Switzerland) using the Haigis formula and the instrument's optimized constants for the particular IOL. All surgeries were performed by the same surgeon, with the refractive target set as close to plano as possible based on the biometry. Each procedure was video-recorded and the duration of the procedure was documented from the recordings.

All patients in the control group underwent the same cataract surgery procedure from the same surgeon, with the same regime and with the same type of IOL, but without the implantation of the new endocapsular device.

### Evaluation

Patients in the experimental group were followed for at least 12 months postoperatively, with evaluations scheduled at 1 day, 1 week, and 1, 3, 6, and 12 months. At each follow-up visit, patients underwent a thorough ophthalmic examination including measurement of uncorrected distance visual acuity (using a tumbling E decimal chart), corrected distance visual acuity (CDVA, using a tumbling E decimal chart, subjective refraction), refraction (both auto and subjective manifest refraction), IOP (Goldmann applanation tonometry, Shin-Nippon, Kagoshima, Japan), optical biometry (LS 900 Lenstar, Haag-Streit, also used preoperatively for IOL calculation), and endothelial cell density (EM-300, Tomey Corporation, Nagoya, Japan). Cataract grading based on the Lens Opacities Classification System III was also performed during slit lamp examination. In addition, anterior and posterior segment optical coherence tomography (OCT) (Spectralis, Heidelberg Engineering, Heidelberg, Germany), Scheimpflug imaging (Oculus Pentacam AXL, Oculus, Menlo Park, CA), and wavefront analysis and corneal Topography (iTrace, Tracey Technologies, Houston, TX) were performed. Anterior chamber depth (ACD) values were calculated using a custom algorithm in MATLAB that analyzed the Scheimpflug images and manually calculated the distance between the posterior cornea surface and the anterior IOL surface. Finally, intraoperative or postoperative complications and PCO were documented. The presence of PCO was evaluated under slit-lamp examination using retroillumination slit-lamp photographs taken after pupil dilation using tropicamide 1.0%.

The primary safety endpoint was the incidence of intraoperative and postsurgical AEs, including their seriousness and causal relationship with the use of the investigational device as documented by the surgeon. The primary efficacy endpoint was the incidence of PCO postoperatively at 12 months. Secondary measurements of the investigation were (a) the assessment of changes of spherical equivalent (SE), (b) the assessment of change of CDVA, (c) the postoperative changes of ACD, and (d) the assessment of changes of IOP.

Beside the preoperative documentation of the control group similar to the experimental group, the incidence rate of PCO in the control group was documented only at 1 year postoperatively.

### Statistical Analysis

Descriptive statistics were presented for all available data by timepoint. Continuous data were conveyed by mean ± standard deviation and categorical data by percentage. Quantitative variables were checked regarding their distribution. Toward this end, both statistical testing (i.e., Kolmogorov–Smirnov test) and graphical approaches (i.e., histograms, Q–Q plots) were implemented, where appropriate. For normally distributed data, parametric tests (i.e., matched paired *t* test) were used for between timepoint comparisons. Non-normally distributed data were compared between timepoints using non-parametric statistical testing (i.e., Wilcoxon signed-rank test). For the comparison of qualitative variables, the χ^2^ test, McNemar test, and Fisher's exact test were implemented, accordingly. A two-sided *P* value of less than 0.05 was considered statistically significant. The analysis was performed using IBM SPSS Statistics (Version 29.0, IBM, Armonk, NY) or RStudio (Version 2024.04.1+748) which is based on R version 4.4.0. For the proper handling of data in statistical analysis, uncorrected distance visual acuity and CDVA were converted to logarithm of the minimum angle of resolution (logMAR) units.^26^

## Results

In total, 121 patients who were screened and met the inclusion criteria were enrolled in the investigation. Of these, 120 patients with a median cataract grade of N2 (interquartile range, N1–N2) had no major protocol deviations and were used for the analysis. Overall, 114 patients (94.2%) attended all study visits. Preoperative and intraoperative characteristics are summarized in Table 1.

**Table 1.** Preoperative and Intraoperative Characteristics of the Experimental Group

Variable	Mean	Median	SD	Minimum	Maximum
Age (years)	63.61	63.90	8.26	42.64	88.08
SE–autorefraction (D)	−1.60	−1.12	4.04	−15.25	12.00
SE–subjective refraction (D)	−1.62	−1.19	3.04	−10.75	4.75
CDVA (logMAR)	0.36	0.30	0.26	0.00	1.30
ACD (mm)	3.96	3.91	0.55	2.55	6.03
ECD (cells/mm^2^)	2408.29	2439.50	290.80	1294.00	2973.00
IOP (mm Hg)	12.04	12.00	2.37	8.00	21.60
IOL power (D)	21.25	21.50	2.57	15.00	25.50
Sex, n (%)	72 (60) Female		48 (40) Male		
Operated eye, n (%)	74 (61.7) OD		46 (38.3) OS		

SD, standard deviation.

In the control group, 46 of the 100 screened patients from the same clinic met the inclusion and exclusion criteria. The control group had a mean age of 68.33 ± 7.53 years old and a mean preoperative SE of −2.63 ± 6.08 D. Compared with the experimental group, age was significantly higher (*P* < 0.05), whereas SE was not (*P* = 0.34). The mean IOL dioptric power was 21.18 ± 3.36 D in the control group, with no significant difference from the experimental group (*P* = 0.96).

A total of 16 AEs were reported in 12 patients (9.9%; 5 male and 7 female), with 56.3% occurring in the right eye. The most common AE was macular edema (seven events) detected by OCT. Among these, five cases were classified as cystoid macula edema, and the remaining two represented mild, subclinical macular thickening, identified on OCT thickness/deviation map without cystoid changes or clinical impact. The percent change in the average central (circle diameter 1 mm) macula thickness from preoperative values ranged from 25.62% to 59.92% in cystoid macula edema cases and from 15.19% to 25.10% in noncystoid cases. All cases resolved, with percent changes below 14.60% for cystoid macula edema (on average, 8.79%) and below 4.45% for on-cystoid cases (on average, 3.70%). Other AEs included elevated IOP (*n* = 2), and single events of retinal detachment, vitreous hemorrhage with posterior vitreous detachment, retinal break, iris patch, corneal degeneration, corneal edema, and IOL dislocation. All AEs were finally resolved and were deemed by the surgeon as unrelated to the investigational device (see Supplementary Table S1 for a more comprehensive list of AEs). The surgery was well-tolerated by both the surgeon and the patient with a recorded mean implantation time for the endocapsular device of 1 minute 42 seconds (standard deviation, 1 minute 36 seconds). Four deaths occurred during follow-up (3 female, 1 male; mean age, 75.01 ± 5.52 years), none of which were related to the study.

Faint PCO was observed only in one patient in the experimental group from 1 day to 12 months of follow-up, without affecting the patient's visual acuity. In the control group, the PCO incidence at 12 months was 13.0%, with three cases requiring Nd:YAG laser treatment.

**Figure 3.**
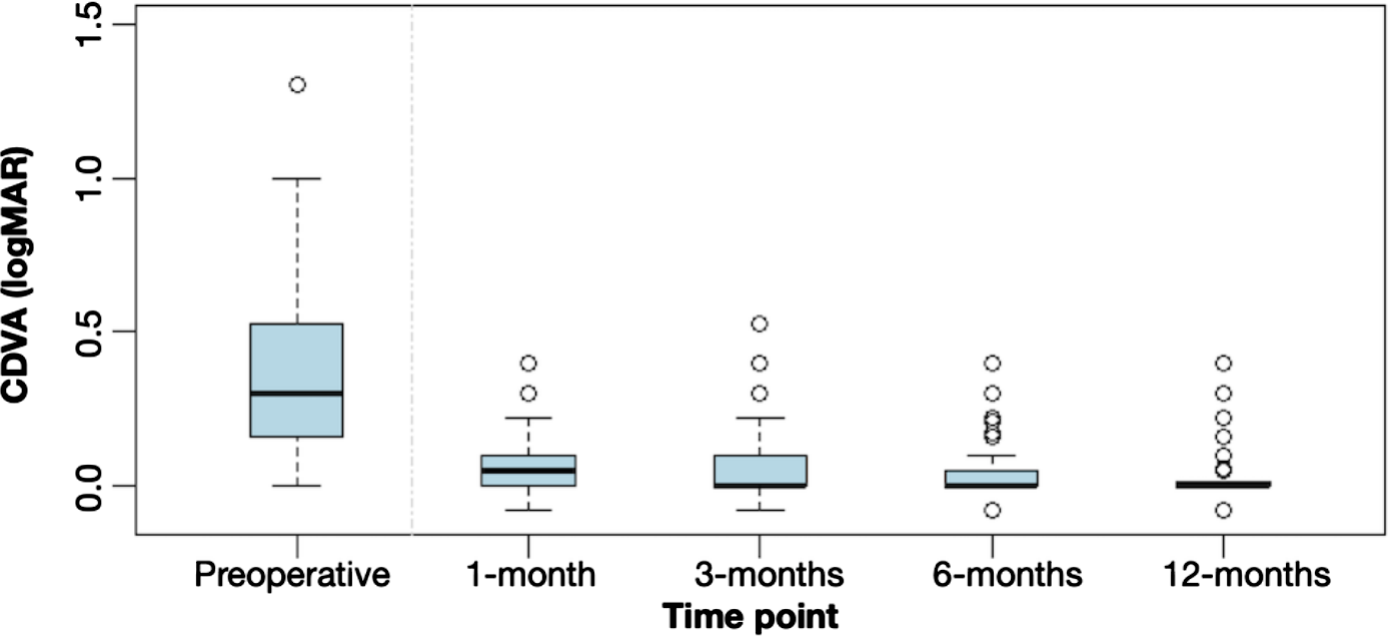
CDVA in logMAR over time.

The mean value for CDVA in the experimental group was 0.08 ± 0.10, 0.06 ± 0.10, 0.04 ± 0.08, and 0.03 ± 0.07 logMAR at 1 month, 3 months, 6 months, and 1 year postoperatively, respectively (Fig. 3). Statistically significant improvements were observed at 3, 6, and 12 months vs 1 month (*P* ≤ 0.001). Differences between 3 months vs. 12 months (*P* < 0.001) and 6 months vs. 12 months (*P* = 0.010) were also significant, whereas the 3 months vs. 6 months comparison approached significance (*P* = 0.050). The mean CDVA differences across timepoints ranged from 0.01 to 0.04 logMAR (Table 2).

**Table 2.** Change From 1, 3, and 6 Months Postoperatively in CDVA (logMAR)

Timepoints	Mean	Median	SD	Min	Max	*P* Value^*^	95% CI^†^
1–3 months	0.02	0.00	0.07	−0.21	0.30	**0.001**	0.023 to 0.076
1–6 months	0.03	0.00	0.07	−0.21	0.30	**<0.001**	0.035 to 0.072
1–12 months	0.04	0.00	0.08	−0.21	0.30	**<0.001**	0.053 to 0.100
3–6 months	0.01	0.00	0.06	−0.18	0.36	0.050	−0.000 to 0.041
3–12 months	0.02	0.00	0.07	−0.18	0.37	**<0.001**	0.027 to 0.067
6–12 months	0.01	0.00	0.05	−0.09	0.24	**0.010**	0.011 to 0.052

CI, confidence interval.

* Results from Wilcoxon signed-rank test. Statistically significant *P* values are presented in bold.

† The 95% CI for the median of the difference.

**Figure 4.**
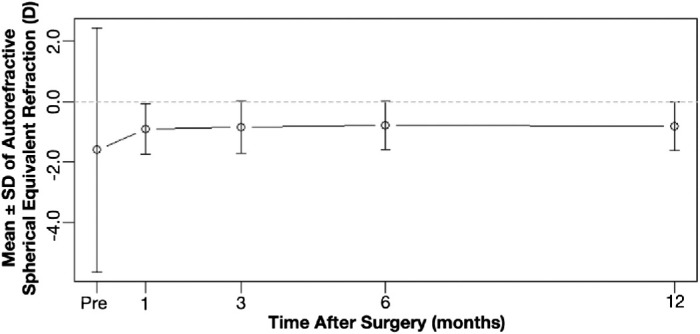
Stability of SE autorefraction over time.

The mean value for autorefractive SE in the experimental group was −0.90 ± 0.84 D (minimum, −2.75 D; maximum, 1.88 D), −0.84 ± 0.86 D (minimum, −3.12 D; maximum, 1.38 D), −0.78 ± 0.80 D (minimum, −3.00 D; maximum, 1.62 D), and −0.81 ± 0.8 D (maximum, −3.12 D; minimum, 1.88 D) at 1 month, 3 months, 6 months, and 1 year postoperatively, respectively (Fig. 4). Significant changes were observed from 1 to 6 months (Δ = −0.11 ± 0.44 D; *P* = 0.012) and from 1 to 12 months (Δ = −0.11 ± 0.44 D; *P* = 0.019); changes beyond 3 months were not significant (*P* ≥ 0.207), indicating stability by month 3 (Table 3). Subjective SE followed similar trends but are not presented herein for brevity.

**Table 3.** Change From 1, 3, and 6 Months Postoperatively in Autorefractive SE (D)

Timepoints	Mean	Median	SD	Minimum	Maximum	*P* Value^*^	95% CI^†^
1–3 months	−0.06	0.00	0.46	−1.38	1.50	0.242	−0.125 to 0.062
1–6 months	−0.11	−0.12	0.44	−1.12	1.00	**0.012**	−0.250 to −0.000
1–12 months	−0.11	−0.12	0.44	−1.12	0.88	**0.019**	−0.188 to −0.000
3–6 months	−0.04	0.00	0.39	−1.12	1.25	0.294	−0.125 to 0.062
3–12 months	−0.06	0.00	0.40	−1.50	0.88	0.207	−0.187 to 0.062
6–12 months	0.00	0.00	0.33	−1.12	1.00	0.788	−0.063 to 0.063

* Results from Wilcoxon signed-rank test. Statistically significant *P* values are presented in bold.

† The 95% CI for the median of the difference.

**Figure 5.**
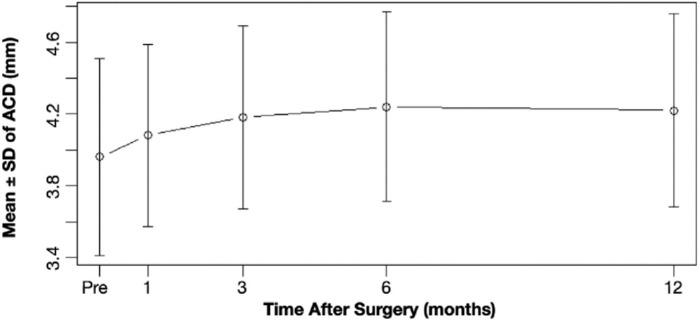
Stability of ACD over time.

The mean value for ACD was 4.08 ± 0.51 mm (minimum, 2.99 mm; maximum, 5.71 mm), 4.18 ± 0.51 mm (minimum, 2.76 mm; maximum, 5.60 mm), 4.24 ± 0.53 mm (minimum, 2.86 mm; maximum, 5.70 mm), and 4.22 ± 0.54 mm (minimum, 2.76 mm; maximum, 5.88 mm) at 1, 3, 6, and 12 months after cataract surgery, respectively (Fig. 5). Significant changes were observed from 1 month to 3, 6, 12 months (Δ = −0.09 ± 0.016 mm, Δ = −0.13 ± 0.18 mm, and Δ = −0.13 ± 0.21 mm, respectively; *P* < 0.001). Comparisons between 3 and 6 months (Δ = −0.05 ± 0.12 mm; *P* < 0.001) and between 3 and 12 months (Δ = −0.04 ± 0.16 mm, *P* = 0.033) were also significant. No significant difference was found between 6 and 12 months (Δ = 0.00 ± 0.18 mm, *P* = 0.570) (Table 4).

**Table 4.** Change From 1, 3, and 6 Months Postoperatively in ACD (mm)

Timepoints	Mean	Median	SD	Minimum	Maximum	*P* Value^*^	95% CI^†^
1–3 months	−0.09	−0.06	0.16	−0.63	0.24	**<0.001**	−0.103 to −0.043
1–6 months	−0.13	−0.12	0.18	−0.67	0.30	**<0.001**	−0.156 to −0.090
1–12 months	−0.13	−0.11	0.21	−0.83	0.31	**<0.001**	−0.157 to −0.079
3–6 months	−0.05	−0.06	0.12	−0.46	0.39	**<0.001**	−0.072 to −0.033
3–12 months	−0.04	−0.03	0.16	−0.80	0.34	**0.033**	−0.061 to −0.002
6–12 months	0.00	0.02	0.18	−0.86	0.54	0.570	−0.038 to 0.022

* Results from Wilcoxon signed-rank test. Statistically significant *P* values are presented in bold.

† The 95% CI for the median of the difference.

**Figure 6.**
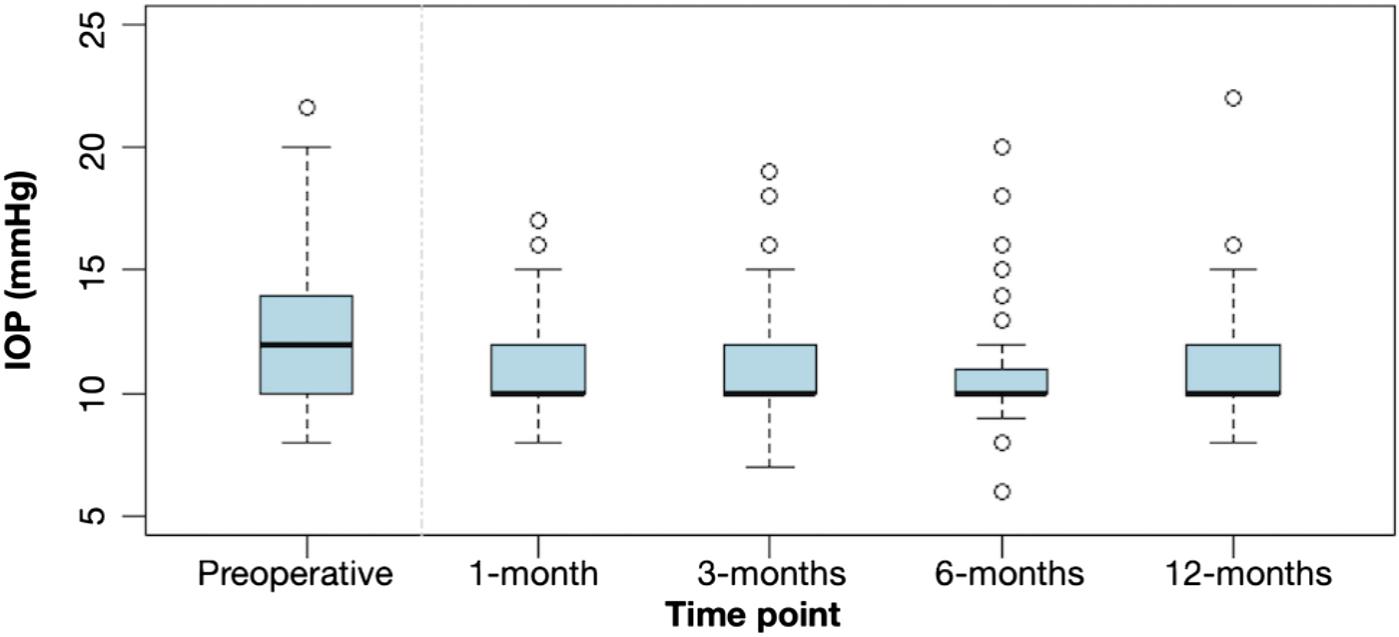
IOP over time. The patient appearing as an outlier with high IOP is the same in all intervals, including the preoperative measurement.

The mean value for IOP in the experimental group was 11.24 ± 2.01 mm Hg, 11.06 ± 1.79 mm Hg, 10.67 ± 1.89 mm Hg and 11.23 ± 2.03 mm Hg at 1 month, 3 months, 6 months, and 1 year postoperatively, respectively (Fig. 6). Compared with preoperative values, the IOP decreased on average postoperatively (Table 5). Only two patients recorded an IOP of greater than 21 mm Hg—one at 1 month and one at 12 months, both isolated findings. It is noteworthy that only one patient who had normal preoperative IOP presented with an IOP of greater than 21 mm Hg at 12 months postoperatively.

**Table 5.** Change From Preoperative and 1, 3, and 6 Months Postoperatively in IOP (mm Hg)

Timepoints	Mean	Median	SD	Minimum	Maximum	*P* Value^*^	95% CI^†^
Pre–1 month	0.73	0.00	2.62	−6.00	8.00	0.015	0.000 to 1.500
Pre–3 months	0.85	0.00	2.82	−8.00	10.60	0.002	0.000 to 1.500
Pre–6 months	1.34	1.00	2.56	−6.00	12.60	<0.001	0.500 to 2.000
Pre–12 months	0.83	0.00	2.72	−6.00	10.00	0.002	1.500 to 3.000
1–3 months	0.00	0.00	1.98	−7.00	7.00	0.783	0.500 to 2.000
1–6 months	0.45	0.00	2.46	−10.00	7.00	0.027	−0.500 to 1.000
1–12 months	−0.07	0.00	2.52	−12.00	6.00	0.990	0.000 to 2.000
3–6 months	0.45	0.00	2.05	−8.00	9.00	0.008	−0.500 to 0.500
3–12 months	−0.22	0.00	2.65	−10.00	9.00	0.360	0.000 to 2.000
6–12 months	−0.54	0	2.31	−6	6.0	0.002	−1.500 to 0.500

Pre, preoperative value.

* Results from Wilcoxon signed-rank test. Statistically significant *P* values are presented in bold.

† The 95% CI for the median of the difference.

## Discussion

Since the introduction of CTRs and associated endocapsular devices, cataract surgery technology has seen substantial improvements. These innovations have primarily aimed to optimize optical outcomes, postoperative visual acuity, and patient satisfaction. However, limited attention has been given to the anatomical restoration of the capsular bag and to the development of more advanced endocapsular devices that prevent postoperative capsular contraction. As such and until the present, current use of CTRs’ and associated endocapsular devices is largely restricted to patients with weak zonular fibers, and more advanced IOLs and capsular devices that keep the bag open have remain underused. To the authors’ best knowledge, this study is the first clinical cohort investigation aiming to evaluate the safety and efficacy of a new endocapsular device designed to preserve capsular shape and endocapsular space in lens extraction surgeries and to reduce the incidence of PCO for at least 12 months postoperatively.

The primary safety endpoint of this study was the incidence of AEs. The study demonstrated that implantation of the new endocapsular device is both feasible and safe, with no serious AEs or device adverse effects. The implantation introduced minimal added surgical time and was associated with a short learning curve. The reported AEs were consistent with those commonly seen in routine phacoemulsification surgery.^10^^,^^27^^–^^31^ Intraoperative complications in cataract surgery have a reported rate of 4.1%, retinal detachment rates vary from 0.9% to 1.79%, and elevated IOP occurs in 2.3% to 8.9% of cases. The incidence of vitreous hemorrhage after cataract surgery varies widely, from 0.45% to 8.22%, whereas posterior vitreous detachment within 1 year is common, with one study finding it in 58.7% of patients without preexisting PVD. IOL dislocation is reported in 0.2% to 1.7% of cases, and persistent corneal edema occurs in 0.5% to 1.0%.

Macular edema remains the most common postoperative complication, with incidence rates ranging from 0.2% to 20.0% in different studies.^29^^,^^32^^–^^34^ Even though our reported incident rate of macula edema in the order of 5.83% (4.17% cystoid and 1.67% for noncystoid) is higher than the 1.17% rate reported by Chu et al. (2016),^32^ a direct comparison (and in this case from a database) is inappropriate owing to differences in populations and diagnostic criteria. In the study by Chu et al. (2016), the presence of postoperative macular edema was obtained from a recorded clinical finding or diagnosis of macular edema or, for eyes from patients with diabetes, newly recorded diagnosis of cystoid macular edema or clinically significant macular edema, and imaging (OCT or angiography) was inconsistently used, potentially under-reporting cases. In contrast, our study used postoperative OCT systematically, allowing for more accurate detection. Prior studies confirm that the use of OCT or angiography increases macular edema detection rates significantly, often ranging from 4% to 11% and up to 41%.^33^^–^^34^ Therefore, the rate of macula edema (cystoid and noncystoid) observed in our study is consistent with the literature when comparable diagnostic modalities are used.

It remains unclear whether preserving the capsular shape influences macular edema formation through other signaling factors as compared with common cataract surgery. Currently, it is believed that manipulation within the anterior chamber of the iris leads to the production of inflammatory mediators that diffuse posteriorly into the vitreous and toward the retina to cause a disruption of the blood-retinal barrier.^35^^–^^37^ Because this new device does not fundamentally alter intraocular manipulation (beyond a brief addition to surgical time), any such influence would need to be examined in future nonhuman studies.

Although the overall AE rate of 9.9% in our study appears to be higher than cumulative rates reported in the literature, the incidence of each individual AE aligns with previously published data. The apparent discrepancy likely reflects differences between clinically based reporting and combined clinical/instrument-based surveillance, as well as variation in event severity categorization. Severe AEs—typical those associated with vision loss—are relatively uncommon, whereas nonsevere AEs that resolve without long-term impact are reported more variably. As a result, direct comparisons across studies are difficult and it remains unclear whether the device contributed to any observed AEs through additional intraoperative manipulation or unique postoperative effects. Larger, controlled studies will be necessary to more definitely assess the influence of the device on AE rates.

The primary efficacy endpoint was the incidence rate of PCO at 12 months. Only one case of PCO was observed, representing a significantly lower incidence than typically reported. Literature on standard phacoemulsification procedures without the use of an endocapsular device report PCO rates of at least 11.8% at the 1-year follow-up,^38^^–^^41^ consistent with rates observed in our control group. Results from the Academy IRIS Registry revealed a 28% PCO rate within 1 year after cataract surgery (in 89,947 eyes), with 10% requiring Nd:YAG laser capsulotomy.^42^ In this same study, the rate of Nd:YAG laser capsulotomy at 1 year in patients implanted with the Technis monofocal IOL was 8.1%. Similarly, early-onset PCO (within 3 months) has been reported in up to 29.93% of eyes after standard cataract surgery.^43^ Even studies using endocapsular devices reported the PCO incidence over 2 years,^14^ while the pilot study of Pallikaris et al.^24^ (2016) using a new design of a thick endocapsular open ring showed mild PCO in 3 of 17 eyes over 3 years. Direct comparisons with more advanced experimental devices are limited owing to differences in study design, duration, and use in nonhuman eyes.^18^^–^^23^

Regarding secondary measurements, results on SE and ACD suggest stable IOL positioning up to 12 months postoperatively. The median SE difference from the 1-month baseline was −0.12 D at 6 and 12 months—statistically significant but below the 0.25 D threshold for clinical relevance. Additionally, 70% to 75% of patients had SE values within ±0.50 D of their 1-month measurements at all follow-ups, and approximately 80% of patients had SE values within ±0.50 D of their 3-month measurements at all follow-ups, aligning with established guidelines for refractive stability.^44^^–^^45^

Likewise, ACD values also stabilized by 6-months, with mean changes ranging from −0.04 to −0.13 mm compared with 1-month postoperatively. Given that a 1-mm change in ACD can induce 0.32 to 1.44 D of refractive shift (1.24 D for a 20 D IOL according to our own observations),^46^^–^^47^ a change of 0.196 mm or less causes a refractive change of less than 0.25 D and was observed in 60% to 80% of patients, indicating consistent IOL positioning. It is noteworthy that a myopic refractive surprise was observed in SE and is consistent with smaller post-ACD values (mean, 4.22 mm at 12 months) as compared with a nominal average value of approximately 4.5 mm implied in many lens calculation formulas. Some CTR studies report no significant effect on refractive stability or optical outcomes.^48^^–^^49^

The better outcomes of the new endocapsular device as compared with most commonly use CTR devices may be attributed to the significant differences between the two, essentially, different modalities. The material of the existing CTRs is polymethyl methacrylate with an average reported diameter in between 12.0 mm and 14.5 mm (greater than that of a capsular bag). They function by applying centrifugal force to the equator of the bag and aim to redistribute forces from intact zonules to buttress areas of absent weak zonules. In contrast, the new device is a closed thicker ring (9.80 mm in diameter, 1.84 mm thick, 32.5 mg weight) designed to maintain capsular shape and intracapsular space improving IOL stability and potentially inhibiting cell migration through more effective peripheral stretching and barrier formation. Its density, being only slightly greater than water, results in a weight far below that of the crystalline lens (on average approximately 250 mg, which increases as the cataract progresses owing to accumulation of proteins),^50^ suggesting minimal gravitational stress. Despite this, long-term studies are required to confirm its long-term effect on the capsule owing to its weight.

With respect to CDVA, the mean difference ranged from 0.01 to 0.04 logMAR at all timepoints, being markedly lower than the clinically meaningful change of 0.1 of letter size progression in the logMAR chart. Similar visual acuity values have been reported in the literature.^29^ Using the aspheric Tecnis Z9000 IOL, mean CDVA has been reported to be −0.053 logMAR at 2 to 3 months after surgery,^51^ whereas with the Tecnis ZB00 the mean CDVA has been shown to range from −0.06 to 0.89 logMAR.^52^^–^^53^

This study has limitations, including the relatively short follow-up period, which may not fully capture long-term PCO development, and a small control group. The slight age difference between groups may introduce bias, although PCO is typically more prevalent in younger patients, in contrast with our findings.^46^ Additionally, the retrospective design of the control group limited our ability to directly compare AEs, allowing only for direct assessment of PCO incidence at 12 months. Nevertheless, although reported PCO rates at 12 months vary widely in the literature, AE data after cataract surgery are generally well-documented. Moreover, variability in results tends to be lower in studies using similar evaluation methods, such as OCT. Therefore, a comparison of our AE rates with those reported in the literature is likely valid and may support more robust conclusions in this regard, in contrast with the interpretation of PCO rates when compared only with results in the literature.

A wide range of refractive surprise was observed, likely owing to interindividual anatomical variations and limitations in current IOL power calculation formulas. Future studies should incorporate more comprehensive biometric data (i.e., white to white, sulcus to sulcus, magnetic resonance imaging data) to refine IOL calculations when using the device. Correlation of these measurements with the final refractive surprise of each patient could provide more insight on this large range of refractive surprise and a more suitable IOL calculation method to be used in conjunction with the new endocapsular device.

Overall, and in agreement with the previous case report,^25^ this study suggests that the new endocapsular device preserves posterior capsular transparency more effectively as compared with other technologies and supports IOL stability, at least up to 12 months after the surgery. These findings reinforce the general belief that endocapsular devices that keep the bag open decrease the incidence of PCO. More data are needed to determine if A-constant adjustments or alternative IOL formulas are required to also provide refractive predictability. Alternatively, more studies may be required to decide on whether a supplementary device needs to be developed to accommodate for the anatomical interpatient variability of the lens. Similar to CTRs and existing endocapsular devices, the size of the capsular bag may dictate variations on the dimension of the new device; it is well-known that capsular bag dimensions correlate well with globe axial length and cornea diameter. Beyond PCO prevention and lens stabilization, we believe that the new endocapsular device may also help to prevent forward movement of the capsule or the vitreous and reduce peripheral light rays—potentially addressing night dysphotopsia—but these hypotheses require further investigation.

## Supplementary Material

Supplement 1

Supplement 2

## Supplementary Material

Supplementary Video S1. Representative video of the procedure whereby the new endocapsular device is implanted before IOL implantation.

## References

[1] Steinberg EP, Javitt JC, Sharkey PD, et al. The content and cost of cataract surgery. *Arch Ophthalmol* 1993; 111(8): 1041–1049. 10.1001/archopht.1993.01090080037016. 8352686

[2] Allen D, Vasavada A. Cataract and surgery for cataract. *BMJ* 2006; 333: 128. 10.1136/bmj.333.7559.128. 16840470 PMC1502210

[3] Brenner LF, Nistad K, Schonbeck U. Rethinking presbyopia: results of bilateral refractive lens exchange with trifocal intraocular lenses in 17 603 patients. *Br J Ophthalmol*. 2023; 107(7): 912–919. 10.1136/bjophthalmol-2021-319732. 35110276

[4] Alio JL, Plaza-Puche AB, Férnandez-Buenaga R, Pikkel J, Maldonado M. Multifocal intraocular lenses: an overview. *Surv Ophthalmol*. 2017; 62(5): 611–634. 10.1016/j.survophthal.2017.03.005. 28366683

[5] Maedel S, Evans JR, Harrer-Seely A, Findl O. Intraocular lens optic edge design for the prevention of posterior capsule opacification after cataract surgery. *Cochrane Database Syst Rev*. 2021; 8: CD012516. 10.1002/14651858.CD012516.pub2. 34398965 PMC8406949

[6] Nagy Z, Takacs A, Filkorn T, Sarayba M. Initial clinical evaluation of an intraocular femtosecond laser in cataract surgery. *J Refract Surg*. 2009; 25(12): 1053–1060. 10.3928/1081597X-20091117-04. 20000286

[7] Fong CS, Mitchell P, Rochtchina E, Cugati S, Hong T, Wang JJ. Three-year incidence and factors associated with posterior capsule opacification after cataract surgery: The Australian Prospective Cataract Surgery and Age-related Macular Degeneration Study. *Am J Ophthalmol*. 2014; 157(1): 171–179.e1. 10.1016/j.ajo.2013.08.016. 24112632

[8] Kanclerz P, Yildirim TM, Khoramnia R. A review of late intraocular lens opacifications. *Curr Opin Ophthalmol*. 2021; 32(1): 31–44. 10.1097/ICU.0000000000000719. 33165018

[9] Moreno-Montañés J, Sánchez-Tocino H, Rodriguez-Conde R. Complete anterior capsule contraction after phacoemulsification with acrylic intraocular lens and endocapsular ring implantation. *J Cataract Refract Surg*. 2002; 28(4): 717–719. 10.1016/s0886-3350(01)01231-7. 11955919

[10] Subasi S, Yuksel N, Karabas VL, Yilmaz Tugan B. Late in-the-bag spontaneous IOL dislocation: risk factors and surgical outcomes. *Int J Ophthalmol*. 2019; 12(6): 954–960. 10.18240/ijo.2019.06.12. 31236352 PMC6580206

[11] Behndig A, Montan P, Stenevi U, Kugelberg M, Zetterström C, Lundström M. Aiming for emmetropia after cataract surgery: Swedish National Cataract Register study. *J Cataract Refract Surg*. 2012; 38(7): 1181–1186. 10.1016/j.jcrs.2012.02.035. 22727287

[12] Karahan E, Er D, Kaynak S. An overview of Nd:YAG laser capsulotomy. *Med Hypothesis Discov Innov Ophthalmol*. 2014; 3(2): 45–50. PMID: 25738159; PMCID: PMC4346677. 25738159 PMC4346677

[13] Nagamoto T, Bissen-Miyajima H. A ring to support the capsular bag after continuous curvilinear capsulorhexis. *J Cataract Refract Surg*. 1994; 20(4): 417–420. 10.1016/s0886-3350(13)80177-0. 7932131

[14] Hara T, Hara T, Narita M, Hashimoto T, Motoyama Y, Hara T. Long-term study of posterior capsular opacification prevention with endocapsular equator ring in humans. *Arch Ophthalmol*. 2011; 129(7): 855–863. 10.1001/archophthalmol.2011.38. 21402979

[15] Trikha S, Agrawal S, Saffari SE, Jayaswal R, Yang YF. Visual outcomes in patients with zonular dialysis following cataract surgery. *Eye*. 2016; 30(10): 1331–1335. 10.1038/eye.2016.108. 27285326 PMC5129851

[16] Rai G, Sahai A, Kumar PR. Outcome of capsular tension ring (CTR) implant in complicated cataracts. *J Clin Diagn Res*. 2015; 9(12): NC05–NC07. 10.7860/JCDR/2015/10425.6999. PMC4717763 26816928

[17] Gimbel HV, Condon GP, Kohnen T, Olson RJ, Halkiadakis I. Late in-the-bag intraocular lens dislocation: incidence, prevention, and management. *J Cataract Refract Surg*. 2005; 31(11): 2193–2204. 10.1016/j.jcrs.2005.06.053. 16412938

[18] Werner l, Pandey SK, Izak AM, et al. Capsular bag opacification after experimental implantation of a new accommodating intraocular lens in rabbit eyes. *J Cataract Refract Surg*. 2004; 30(5): 1114–1123. 10.1016/j.jcrs.2003.09.044. 15130653

[19] Ossma IL, Galvis A, Vargas LG, Trager MJ, Vagefi MR, McLeod SD. Synchrony dual-optic accommodating intraocular lens. Part 2: pilot clinical evaluation. *J Cataract Refract Surg*. 2007; 33(1): 47–52. 10.1016/j.jcrs.2006.08.049. 17189792

[20] Kavoussi SC, Werner L, Fuller SR, et al. Prevention of capsular bag opacification with a new hydrophilic acrylic disk-shaped intraocular lens. *J Cataract Refract Surg*. 2011; 37(12): 2194–2200. 10.1016/j.jcrs.2011.05.049. 22108114

[21] Floyd AM, Werner L, Liu E, et al. Capsular bag opacification with a new accommodating intraocular lens. *J Cataract Refract Surg*. 2013; 39(9): 1415–1420. 10.1016/j.jcrs.2013.01.051. 23831157

[22] Eldred JA, Spalton DJ, Wormstone M. An in vitro evaluation of the Anew Zephyr open-bag IOL in the prevention of posterior capsule opacification using a human capsular bag model. *Invest Ophthalmol Vis Sci*. 2014; 55(11): 7057–7064. 10.1167/iovs.14-15302. 25237161

[23] Slutzky L, Kleinmann G. Further enhancement of intraocular open-capsule devices for prevention of posterior capsule opacification. *Transl Vis Sci Technol*. 2018; 7(1): 21. 10.1167/tvst.7.1.21. PMC5829951 29497583

[24] Pallikaris IG, Stojanovic NR, Ginis HS. A new endocapsular open ring for prevention of anterior and posterior capsule opacification. *Clin Ophthalmol*. 2016; 10: 2205–2212. 10.2147/OPTH.S106770. 27843291 PMC5098592

[25] Elmassry A, Sahin O, Leonidou L, et al. Preservation of capsular transparency and geometrical consistency in cataract surgery using a novel intracapsular ring. *J Clin Res Ophthalmol*. 2020; 7(2): 101–104. 10.17352/2455-1414.000082.

[26] Holladay JT . Visual acuity measurements. *J Cataract Refract Surg*. 2004; 30(2): 287–290. 10.1016/j.jcrs.2004.01.014. 15030802

[27] Chan E, Mahroo OAR, Spalton DJ. Complications of cataract surgery. *Clin Exp Optom*. 2010; 93(6): 379–389. 10.1111/j.1444-0938.2010.00516.x. 20735786

[28] Stein JD . Serious adverse events after cataract surgery. *Curr Opin Ophthalmol*. 2012; 23(3): 219–225. 10.1097/ICU.0b013e3283524068. 22450221 PMC3777802

[29] Day AC, Donachie PHJ, Sparrow JM, Johnston RL. The Royal College of Ophthalmologists’ National Ophthalmology Database study of cataract surgery: report 1, visual outcomes and complications. *Eye*. 2015*;* 29(4): 552–560. 10.1038/eye.2015.3. 25679413 PMC4816350

[30] Wang J, Su F, Wang Y, Chen Y, Chen Q, Li F. Intra and post-operative complications observed with femtosecond laser-assisted cataract surgery versus conventional phacoemulsification surgery: a systematic review and meta-analysis. *BMC Ophthalmol*. 2019; 19(1): 177. 10.1186/s12886-019-1190-2. 31399070 PMC6688351

[31] Qureshi MH, Steel DHW. Retinal detachment following cataract phacoemulsification—a review of the literature. *Eye*. 2020; 34(4): 616–631. 10.1038/s41433-019-0575-z. 31576027 PMC7093479

[32] Chu CJ, Johnston RL, Buscombe C, Sallam AB, Mohamed Q, Yang YC, United Kingdom Pseudophakic Macular Edema Study Group. Risk factors and incidence of macular edema after cataract surgery: a database study of 81984 eyes. *Ophthalmology*. 2016; 123(2): 316–323. 10.1016/j.ophtha.2015.10.001. 26681390

[33] Lobo C . Pseudophakic cystoid macular edema. *Ophthalmologica*. 2012; 227(2): 61–67. 10.1159/000331277. 21921587

[34] Yonekawa Y, Kim IK. Pseudophakic cystoid macular edema. *Curr Opin Ophthalmol*. 2012; 23(1): 26–32. 10.1097/ICU.0b013e32834cd5f8. 22134362

[35] Benitah NR, Arroyo JG. Pseudophakic cystoid macular edema. *Int Ophthalmol Clin*. 2010; 50(1): 139–153. 10.1097/IIO.0b013e3181c551da. 20057303

[36] Hudes GR, Li WY, Rockey JH, White P. Prostacyclin is the major prostaglandin synthesized by bovine retinal capillary pericytes in culture. *Invest Ophthalmol Vis Sci*. 1988; 29(10): 1511–1516. PMID: 3049428 3049428

[37] Panteleontidis V, Detorakis ET, Pallikaris IG, Tsilimbaris MK. Latanoprost-dependent cystoid macular edema following uncomplicated cataract surgery in pseudoexfoliative eyes. *Ophthalmic Surg Lasers Imaging*. 2010; 9: 1–5. 10.3928/15428877-20100215-91. 20337282

[38] Schaumberg DA, Dana MR, Christen WG, Glynn RJ. A systematic overview of the incidence of posterior capsule opacification. *Ophthalmology*. 1998; 105(7): 1213–1221. 10.1016/S0161-6420(98)97023-3. 9663224

[39] Clark DS . Posterior capsule opacification. *Curr Opin Ophthalmol*. 2000; 11(1): 56–64. 10.1097/00055735-200002000-00009. 10724829

[40] Brézin AP, Labbe A, Schweitzer C, et al. Incidence of Nd:YAG laser capsulotomy following cataract surgery: a population-based nation-wide study - FreYAG1 study. *BMC Ophthalmol*. 2023; 23(1): 417. 10.1186/s12886-023-03134-6. 37845645 PMC10578013

[41] Konopińska J, Młynarczyk M, Dmuchowska DA, Obuchowska I. Posterior capsule opacification: a review of experimental studies. *J Clin Med*. 2021; 10(13): 2847. 10.3390/jcm10132847. 34199147 PMC8269180

[42] Horn JD, Fisher BL, Terveen D, Fevrier H, Merchea M, Gu X. Academy IRIS registry analysis of incidence of laser capsulotomy due to posterior capsule opacification after intraocular lens implantation. *Clin Ophthalmol*. 2022; 16: 1721–1730. 10.2147/OPTH.S358059. 35673348 PMC9167596

[43] Gu X, Chen X, Jin G, et al. Early-onset posterior capsule opacification: incidence, severity, and risk factors. *Ophthalmol Ther*. 2022; 11(1): 113–123. 10.1007/s40123-021-00408-4. 34727350 PMC8770765

[44] Gale RP, Saldana M, Johnston RL, Zuberbuhler B, McKibbin M. Benchmark standards for refractive outcomes after NHS cataract surgery. *Eye*. 2009; 23(1): 149–152. 10.1038/sj.eye.6702954. 17721503

[45] Hahn U, Krummenauer F, Kolbl B, et al. Determination of valid benchmarks for outcome indicators in cataract surgery. *Ophthalmology*. 2011; 118(11): 2105–2112. 10.1016/j.ophtha.2011.05.011. 21856011

[46] Norrby S . Sources of error in intraocular lens power calculation. *J Cataract Refract Surg*. 2008; 34(3): 368–376. 10.1016/j.jcrs.2007.10.031. 18299059

[47] Engren AL, Behndig A. Anterior chamber depth, intraocular lens position, and refractive outcomes after cataract surgery. *J Cataract Refract Surg*. 2013; 39(4): 572–577. 10.1016/j.jcrs.2012.11.019. 23395354

[48] Nistad K, Göransson F, Støle E, Shams H, Gjerdrum B. The use of capsular tension rings to reduce refractive shift in patients with implantation of trifocal intraocular lenses. *J Refract Surg*. 2017; 33(12): 802–806. 10.3928/1081597X-20170829-02. 29227507

[49] Xie T, Liu X, Zhu J, Li X. Effect of capsular tension ring on optical and multifunctional lens position outcomes: a systematic review and a meta-analysis. *Int Ophthalmol*. 2021; 41(12): 3971–3984. 10.1007/s10792-021-01969-w. 34302267

[50] Mohamed A, Augusteyn RC. Human lens weights with increasing age. *Mol Vis*. 2018; 24: 867–xxx. PMCID: PMC6382474. 30820139 PMC6382474

[51] Bellucci R, Scialdone A, Buratto L, et al. Visual acuity and contrast sensitivity comparison between Tecnis and AcrySof SA60AT intraocular lenses: a multicenter randomized study. *J Cataract Refract Surg*. 2005; 31(4): 712–717. 10.1016/j.jcrs.2004.08.049. 15899447

[52] Wahba SS, Riad RF, Morkos FF, Hassouna AK, Roshdy MM. Visual performance of the Tecnis one-piece lens ZCB00. *Clin Ophthalmol*. 2011; 5: 1803–1808. 10.2147/OPTH.S27324. 22267915 PMC3258090

[53] Danzinger V, Schartmuller D, Lisy M, et al. Intraindividual comparison of an enhanced monofocal and an aspheric monofocal intraocular lens of the same platform. *Am J Ophthalmol*. 2024; 261: 95–102. 10.1016/j.ajo.2023.11.006. 37944686

