# Supplementary material for: Safety and Efficacy of a New Endocapsular Device Used in Age-Related Cataract Surgery: Twelve-Month Follow-Up
Source: Transl Vis Sci Technol. 2026 Feb 6;15(2):8. doi: 10.1167/tvst.15.2.8 (PMC12889166; doi:10.1167/tvst.15.2.8)
Supplement: Supplement 1 [file tvst-15-2-8_s001.pdf]

Supplementary Table S1. Listing of the observed AEs in the study.

Gender	Age	Eye	Start date	Serious	Description	Treatment	Outcome	End date	Relationship to the device
M	68.73	OD	10/10/20	N	Iris patch	No treatment applied	Resolved	08/05/21	Not related
M	68.73	OD	12/12/20	N	Corneal Degeneration	No treatment applied	Resolved	08/05/21	Not related
F	69.75	OS	14/03/21	N	Macular edema	14/03/21: Nevanac ED (3 times/day) / RepariL tab (3 times/day)<br>02/05/21 & 05/06/21: Same for 1 more month	Resolved	05/06/21	Not related
M	71.24	OS	04/01/21	Y	Retinal Detachment	17/01/21: Scleral Buckle + gas injection	Resolved	10/04/21	Not related
M	71.24	OS	10/4/21	N	Macular edema	10/04/21: R1 Nevonac (ED, 3 times/day) / R1 Reparil (tab, 2 times/day) / Predforte (ED, 3 times/day)<br>08/05/21 & 08/06/21: Repeat treatment	Resolved	03/10/21	Not related
F	71.83	OS	11/01/21	N	Macular edema	11/01/21: R1 Nevonac (ED, 3 times/day) / R1 Reparil (tab, 2 times/day) / Predforte (ED, 3 times/ day)	Resolved	19/04/21	Not related
F	58.93	OD	20/12/20	Y	High IOP	20/12/20: Twinzol ED (3 times/day) / Alphaganp ED (3 times/day) / Ioprost ED (once daily). Note: CDR (0.7) OCT (RNFL,å Normal). 11/01/21: Same treatment	Resolved	11/01/21	Not related
F	58.93	OD	21/02/21	N	Central macular edema	21/02/21: Add Nevanac (ED)/ Reparil tab<br>08/05/21: R1 Dexaflex (ED)<br>28/04/21: Intravitreal injection of Trimethazone	Resolved	28/06/21	Not related
F	66.4	OS	03/04/21	N	Macular edema	03/04/21: R1 Nevonac (ED, 3 times/day) / R1 Reparil (tab, 2 times/day)	Resolved	26/06/21	Not related
M	68.11	OD	05/12/21	Y	Vitreous hemorrhage with PVD	05/12/21: Alphontern tab 2x3x3 week / Dycinon tab 1x3x3 week	Resolved	10/01/22	Not related
M	68.11	OD	10/01/22	Y	New Retinal break	13/01/21: Cryo and gas injection	Resolved	21/01/22	Not related
M	59.21	OS	21/03/21	N	Macular edema	R1 Nevaxal ED (3 times/day) / R1 Reparil tab (3 times/day)	Resolved	23/05/21	Not related
M	73.23	OD	14/03/21	N	IOL dislocation	22/03/21: IOL repositioning	Resolved	22/03/21	Not related
F	64.24	OD	17/04/21	N	Macular edema	17/04/21: R1 Nevaxal (ED, 3 times/day) / R1 Reparil tab (3 times/day)<br>07/06/21 & 20/09/21: Same treatment for 1 more month	Resolved	20/09/21	Not related
F	53.25	OD	26/04/21	Y	High IOP	26/04/21: Twinzol ED (3 times /day) / Alphaganp ED (3 times/day) / Ioprost ED (once daily)	Resolved	27/06/21	Not related
F	52.37	OS	14/08/21	N	Corneal edema	14/08/21: Optipred 3 times and Twinzol 2 times for 1 month.	Resolved	14/11/21	Not related

Dates stated as DD/MM/YY. Posterior Vitreous Detachment (PVD).
